# Development of the early fetal human thalamus: from a protomap to emergent thalamic nuclei

**DOI:** 10.3389/fnana.2025.1530236

**Published:** 2025-02-07

**Authors:** Maznah Alhesain, Ayman Alzu’bi, Niveditha Sankar, Charles Smith, Janet Kerwin, Ross Laws, Susan Lindsay, Gavin J. Clowry

**Affiliations:** ^1^Newcastle University Biosciences Institute and Centre for Transformative Neuroscience, Newcastle upon Tyne, United Kingdom; ^2^Newcastle University Biosciences Institute and Human Developmental Biology Resource, Newcastle upon Tyne, United Kingdom; ^3^Department of Basic Medical Sciences, Yarmouk University, Irbid, Jordan; ^4^Electron Microscopy Research Services, Newcastle University, Newcastle upon Tyne, United Kingdom

**Keywords:** CNTNAP2, development, diencephalon, human, protomap, thalamus, transcription factor expression

## Abstract

**Introduction:**

Most of what is known about thalamic development comes from rodent studies, however, the increased proportion of human association cortex has co-evolved with increased thalamocortical connectivity. Higher order thalamic nuclei, relaying information between cortical regions and important in higher cognitive function, are greatly expanded.

**Methods:**

This study mapped the emergence of thalamic nuclei in human fetal development (8–16 post conceptional weeks; PCW) by revealing gene expression patterns using in situ hybridization and immunohistochemistry for previously established thalamic development markers.

**Results:**

In the proliferative thalamic ventricular zone, OLIG3 and NR2F1 immunoreactivity marked the extent of the thalamus, whereas PAX6 and NR2F2 were expressed in gradients, suggesting an early protomap. This was also the case for post-mitotic transcription factors *ZIC4*, GBX2, FOXP2 and OTX2 which marked thalamic boundaries but also exhibited opposing gradients with *ZIC4* expression higher anterior/lateral, and GBX2, FOXP2 and OTX2 higher in posterior/medial. Expression patterns became increasingly compartmentalized as development progressed and by 14 PCW recognizable thalamic nuclei were observed with, for instance, the centromedian nucleus being characterized by high FOXP2 and absent GBX2 expression. SP8-like immunoreactivity was expressed in distinct thalamic locations other than the reticular formation which has not been previously reported. Markers for GABAergic neurons and their precursors revealed the location of the prethalamus and its development into the reticular formation and zona incerta. No GAD67+ neurons were observed in the thalamus at 10 PCW, but by 14 PCW the medial posterior quadrant of the thalamus at various levels was infiltrated by GAD67+/ *SOX14*+ cells of presumed pretectal/midbrain origin. We compared expression of the neurodevelopmental disease susceptibility gene *CNTNAP2* to these patterns. It was highly expressed by glutamatergic neurons in many thalamic regions by 14 PCW, sometimes but not always in conjunction with its upstream expression regulator FOXP2.

**Conclusion:**

In human discrete thalamic nuclei exhibiting discrete gene expression patterns emerge relatively early from a protomap of gene expression. The migration of GABAergic neurons into the thalamus occurs over a protracted period, first from the midbrain. Disruption of CNTNAP2 activity and function could be hypothezised to have a variety of effects upon thalamic development.

## Introduction

The vast majority of studies into the development of the thalamus have been in animal models and primarily of first order pathways that transmit sensory information to primary sensory cerebral cortex. However, the greater amount of association cortex found in the human cerebrum, responsible for higher cognitive functioning, has appeared hand in hand with co-evolution of thalamocortical connectivity (Sherman, 2016). Higher order thalamic nuclei such as the mediodorsal nucleus (MD) and the pulvinar, which relay information from one cortical region to another, are greatly expanded in primates, especially human, compared to rodents (Baldwin et al., 2017; Pergola et al., 2018; Homman-Ludiye and Bourne, 2019) and are involved in uniquely human cognitive functions such as lexico-semantic processing of language (Kraut et al., 2003; Van der Werf et al., 2003; Assaf et al., 2006).

According to the widely accepted prosomere model of diencephalic development (Puelles et al., 2013) caudally the first prosomeric domain (p1) gives rise to the pretectum, p2 the thalamus (or dorsal thalamus) and epithalamus and p3 the prethalamus (or ventral thalamus) anteriorly, separated from p2 by the zona limitans intrathalamica (ZLI) an organizer region secreting morphogens including sonic hedgehog (SHH) that facilitate establishment of positional identity (Kiecker and Lumsden, 2004; Nakagawa, 2019; Puelles, 2019). Two thalamic domains are formed; lower levels of SHH result in the production of excitatory glutamatergic neurons in caudal regions of the thalamus, while higher levels induce formation of gamma-aminobutyric acid synthesizing (GABAergic) neurons in the rostral part (Vue et al., 2007; Robertshaw et al., 2013). The rostral part is considerably smaller than the caudal and expresses some (e.g., *GAD* genes, *ASCL1)* but not all genes (e.g., *OLIG3* instead of *OLIG 2*) that the prethalamus expresses (Huerga-Gómez et al., 2023). It is proposed to produce GABAergic neurons for the ventral lateral geniculate nucleus and intergeniculate leaflet (Vue et al., 2007). In this study we generally refer to the caudal part as the thalamus, whilst recognizing the epithalamus as a separate posterior and dorsal domain. Other factors such as fibroblast growth factors and SHH from basal regions are required for maintaining ZLI and for thalamic patterning (Nakagawa, 2019). Our first aim was to examine the expression of key molecules that have been shown to guide the development of the thalamus in animal models. To this end, we studied the expression of a number of transcription factors involved in patterning, including *ZIC4*, GBX2, FOXP2, PAX6, NR2F1, and NR2F2.

*ZIC4* is expressed in p2 (and other dorsal and medial structures) in early developing mouse brain Gaston-Massuet et al., 2005) becoming restricted to specific thalamic nuclei later in development in mouse and marmoset (Horng et al., 2009; Li et al., 2018; Homman-Ludiye et al., 2018). NR2F1 (also known as COUP-TFI) is also expressed throughout p2 during mouse development (Qiu et al., 1994; Liu et al., 2000) and is required for guidance of thalamocortical axon growth (Zhou et al., 1999). On the other hand, *Gbx2* expression in rodents is restricted to thalamocortical projection neurons within p2 along its anterior-posterior axis, as opposed to habenula and prethalamic regions (Bulfone et al., 1993; Chen et al., 2009). All thalamic neurons express *Gbx2* at some point in their development and it is essential for axon outgrowth and pathfinding (Miyashita-Lin et al., 1999; Chatterjee and Li, 2012) and suppression of habenular identity (Chen et al., 2009: Mallika et al., 2015). FOXP2 is expressed in the thalamus in the developing mouse and human (Ferland et al., 2003; Vargha-Khadem et al., 2005). A gradient of expression (higher in the posterior ventral region) in embryonic mouse indicates that FOXP2 is essential for thalamus patterning (Ebisu et al., 2017).

NR2F2 (COUP-TFII) shows relatively reduced expression in the developing mouse thalamus compared to NR2F1 but is strongly expressed in adjacent pretectum and prethalamus (Qiu et al., 1994). PAX6 expression is necessary for the proper maturation of the thalamus (Schmahl et al., 1993; Robertshaw et al., 2013; Caballero et al., 2014; Clegg et al., 2015) and thalamic progenitor cells express it throughout the initial phases of diencephalic development. It is also expressed by progenitor cells of the prethalamus, some of the progeny of which retain expression as post-mitotic neurons (Duan et al., 2013; Caballero et al., 2014).

The diencephalic ventricular zone (VZ) lining the third ventricle contains apical radial glia (Frassoni et al., 2000) that divide asymmetrically to generate post-mitotic neurons (Nakagawa and Shimogori, 2012). These neurons then migrate to the mantle zone where they aggregate eventually forming individual nuclei (Jones and Rubenstein, 2004; Nakagawa and Shimogori, 2012). Similar to the neocortex, there is a thalamic subventricular zone that contains basal progenitors in mouse and human thalamus (Wang et al., 2011; Kim et al., 2023). Excitatory glutamatergic neurons in the thalamus all derive from the ventricular and subventricular zones of p2 (Vue et al., 2007).

GABAergic neurons of the thalamic reticular nucleus, derived from the progenitor zone of p3 (prethalamus; Puelles and Rubenstein, 2003) are proposed to provide the majority of inhibitory drive to the thalamic nuclei In rodent, with very few inhibitory interneurons found within the dorsal thalamic nuclei, with the exception of some visual centers (Ohara et al., 1983; Warren et al., 1994; Arcelli et al., 1997). However, in primates up to 30% of neurons are reported to be GABAergic interneurons in all nuclei (Montero and Zempel, 1986; Hunt et al., 1991) making this a major feature of the evolution of the thalamus. Interneurons migrate into the thalamus at later developmental stages (Jones, 2002) and have been shown to be of predominantly pretectal (p1) and midbrain origin, expressing the transcription factor SOX14 (Jager et al., 2016). Recent studies have shown that GABAergic neurons arising from SOX14+ precursors in mouse are found in all caudal sensory relay nuclei and associated higher order nuclei, however, in marmoset, such cells are more abundant, and also found in anterior, higher order thalamic nuclei (Jager et al., 2021). Other potential sources of thalamic interneurons include the prethalamus (demonstrated in mouse; Jager et al., 2021) and the ganglionic eminences, proposed to be a unique source of interneurons for the human higher order thalamic nuclei (Letinic and Rakic, 2001; Bakken et al., 2021; Kim et al., 2023). A second objective of this study was to throw further light on the origins of GABAergic neurons in the human thalamus.

Some models of psychosis implicate thalamic association nuclei with the pathogenesis of such conditions (Andreasen, 1997; Steullet, 2020; Anticevic and Halassa, 2023). For instance, individuals with psychosis display smaller volumes on average for these nuclei compared to control groups (Huang et al., 2020). Certain nuclei, for instance the medial pulvinar, which forms reciprocal connections with multiple cortical areas and is believed to act as a multimodal, modulatory association center (Homman-Ludiye and Bourne, 2019) are unidentified in rodents (Baldwin et al., 2017; Homman-Ludiye and Bourne, 2019) but implicated in neurodevelopmental diseases of cognition such as schizophrenia (Homman-Ludiye and Bourne, 2019). Post mortem quantitative studies have revealed a decreased neuronal density in the medial pulvinar of schizophrenia patients which, due to the absence of glial scarring, has been attributed to disrupted neurogenesis (Byne et al., 2007). Functional and resting state MRI demonstrate reduced medial pulvinar–temporal lobe connectivity (Cobia et al., 2017) leading to abnormal thalamic activation and cognition.

We hypothesized that potential susceptibility genes for neurodevelopmental disorders (NNDs) may be preferentially expressed in higher order thalamic nuclei. Our third aim was to provide a map of the developing human thalamus in order to locate expression of NND susceptibility genes in early development. We tested this approach by localizing expression of *CNTNAP2*. Bi-allelic mutations in this gene can specifically cause a severe cortical dysplasia focal epilepsy syndrome (Strauss et al., 2006). Genomic rearrangements and point mutations resulting in haploinsufficiency for *CNTNAP2* have been associated with autistic spectrum disorders, schizophrenia and language disorders (Friedman et al., 2008; Rodenas-Cuadrado et al., 2014; Poot, 2015; D’Onofrio et al., 2023; Valeeva et al., 2024) although this is not universally accepted (Toma et al., 2018). It is the largest gene in the human genome, coding for a neurexin-like cell adhesion molecule contactin-associated protein 2 (Nakabayashi and Scherer, 2001) the main function of which is to sequester ion channels at the nodes of Ranvier (Poliak et al., 2003). It is believed to have important roles in development which are less well defined (St George-Hyslop et al., 2022). *CNTNAP2* expression is downregulated by FOXP2, which binds its first intron (Spiteri et al., 2007; Vernes et al., 2007). This study is an early step to understanding how mutations in *CNTNAP2* might affect human thalamic development leading to NNDs.

## Materials and methods

### Human tissue

Human fetal tissue from terminated pregnancies was obtained from the joint MRC/Wellcome Trust-funded Human Developmental Biology Resource (HDBR)^1^ Gerrelli et al., 2015). All tissue was collected with appropriate maternal consent and approval from the Newcastle and North Tyneside NHS Health Authority Joint Ethics Committee. Fetal samples ranging in age from 8 to 16 PCW were used. Ages were estimated from foot and heel to knee length measurements according to Hern (1984). Six samples in total were studied, 2 at 8PCW, 3 at 10 PCW, 1 at 14 PCW, 1 at 15 PCW and 1 at 16 PCW. Samples were dissected, fixed in buffered 4% paraformaldehyde solution and embedded in paraffin and then sectioned according to standard protocols applied by the HDBR staff.^2^

### Immunoperoxidase histochemistry (DAB and Immunofluorescence)

This was carried out on 8 μm thick paraffin sections. Antigen retrieval involved boiling in 10 mM citrate buffer pH6 for 10 min. After washing in Tris buffered saline pH7.6 (TBS), sections were incubated with primary antibody (diluted in 10% normal blocking serum in TBS overnight at 4°C. Details of primary antibodies are found in Table 1. Following 3 washes in TBS, sections were incubated with HRP-conjugated secondary antibody for 30 min (ImmPRESS HRP IgG [Peroxidase] Polymer Detection Kit, Vector Labs) washed and then developed with diaminobenzidine solution (Vector Labs supplied kit) washed, dehydrated, and mounted using DPX (Sigma-Aldrich). For double immunofluorescence, the Tyramide Signal Amplification (TSA) method was used permitting double staining using same species antibodies. At the secondary antibody stage, sections were incubated with HRP-conjugated secondary antibody for 30 min (ImmPRESS HRP IgG [Peroxidase] Polymer Detection Kit, Vector Labs) and then incubated in the dark for 10 minutes with fluorescein tyramide diluted at 1/500 (TSA) fluorescein plus system reagent (Perkin Elmer, Buckingham, United Kingdom) or OPAL 520 diluted 1;700 (Akoya Biosciences, Marlborough MA, United States) leaving fluorescent tags covalently bound to the section. Sections were then boiled in 10 mM citrate buffer pH6 to remove all antibodies and unbound fluorescein then incubated first in 10% normal serum then with the second primary antibody for 2 h at room temperature. Sections were again incubated with HRP-conjugated secondary antibody followed by CY3 tyramide for 10 min ([TSA] CY3 plus system reagent, Perkin Elmer) or OPAL 570 (Akoya Biosciences). Sections were dyed with 4’,6-diamidino-2-phenylindole dihydrochloride (DAPI; Thermo Fisher Scientific) and mounted using Vectashield Hardset Mounting Medium (Vector Labs). Extensive washing of sections was carried out between all incubations (for more details see Alzu’bi et al., 2022).

**TABLE 1:** Details of primary antibodies and RNAScope *in situ* hybridization probes employed.

Antigen	Species	Dilution	Supplier	RRID number	Previous application in human tissue/cells
FOXP2	Mo mAb	1/50	Santa Cruz Heidelberg, Germany.	AB_2721204	Miura et al., 2020
GBX2	Ra pAb	1/500	Proteintech Manchester, UK.	AB_2878896	Alzu’bi et al., 2019
OTX2	Mo mAb	1/200	Santa Cruz.	AB_2921699	Mun et al., 2024
PAX6	Ra pAb	1/1,000	Abcam, Cambridge, UK	AB_2750924	Yamashita et al., 2022
SP8	Ra pAb	1/200	Sigma-Aldrich, Poole, UK.	AB_2682340	https://www.proteinatlas.org/ENSG00000164651-SP8/summary/antibody
CALB1	Ra pAb	1/1,000	Swant, Marly, Switzerland	AB_10000340	Alzu’bi et al., 2019
NR2F1	Mo mAb	1/500	Abcam.	AB_41858	Alzu’bi et al., 2017a
NR2F2	Mo mAb	1/1,000	R&D Systems, Abingdon, UK.	AB_2155627	Alzu’bi et al., 2017b
OLIG2	Ra pAb	1/1,000	Merck/Millipore, Watford, UK.	AB-10141047	Alzu’bi et al., 2017b
OLIG3	Ra pAb	1/100	Abcam	Cat. No. 230626	No previous publications
GAD67	Mo mAb	1/500	Merck Millipore.	AB_2278725	Alzu’bi and Clowry, 2019
Ki67	Mo mAb	1/150	Santa Cruz Biotechnology	AB_627859	Alzu’bi and Clowry, 2019
***ACD Probe***	**Cat No.**	***Gene ID***	**Target region and probe size**	**Accession no**	
*Hs-ZIC4*	525661-C1	84107	2–1724 23zz	NM_001168378	
*Hs-SOX14*	1055251-C1	8403	282–1657 20zz	NM_004189	
*Hs-CNTNAP2*	411341-C1	26047	3556–4477 20zz	NM_014141	

### RNAScope *in situ* hybridization

RNA *in situ* hybridization experiments were performed using the RNAscope^®^ technology, which has been previously described (Wang et al., 2012). Paired double-Z oligonucleotide probes against target RNA for *SOX14, ZIC4*, and *CNTNAP2* were designed and supplied by ACD Bio Techne (Abingdon, United Kingdom) and described in Table 1. The *RNAscope* Reagent Kit (ACD Bio Techne) was used according to the manufacturer’s instructions but with slight modifications. In brief, 8μm-thick paraffin sections were baked on a heating pad for ten minutes at 60°C, dewaxed in Xylene, and then boiled with target retrieval buffer (ACD) for 20 min at 95°C. Protease digestion was carried out at 40°C for 30 min, followed by probe hybridization for 2 hours at 40°C with target probes. The hybridized signals were amplified by a cascade of signal amplification molecules and detected with the RNA*scope* 2.5 HD detection kit (Fast Red). Slides were counterstained with 50% hematoxylin and positive signals showed as red chromogenic dots in the cytoplasm or nucleus. Each sample was quality controlled for RNA integrity with a probe specific to the housekeeping gene *GAPDH*. Negative control background staining was evaluated using a probe specific to the bacterial *dapB* gene (Alzu’bi et al., 2022).

### RNAScope fluorescent *in-situ* hybridization coupled with immunofluorescence

*In situ* hybridization was carried out first as described above except that the hybridized signals were detected with either Cy3 tyramide diluted 1:500 or Opal 570 diluted 1:700. Then Immunofluorescent staining was carried out according to previously described protocols (Alzu’bi et al., 2022) with the antibodies used described in Table 1. Briefly, sections were boiled in 10 mM citrate buffer pH6, followed by incubation with primary antibody (diluted in 10% normal blocking serum in Tris buffered saline [TBS] pH 7.6) overnight at 4°C. Sections were then incubated with HRP-conjugated secondary antibody for 30 min (ImmPRESS HRP IgG [Peroxidase] Polymer Detection Kit, Vector Labs). Signals were detected by incubation with fluorescein tyramide or OPAL 520 as described above. Sections were counterstained with 4′, 6-diamidino-2-phenylindole dihydrochloride (DAPI; Thermo Fisher Scientific, Cramlington, United Kingdom) and mounted using Vectashield Hardset Mounting Medium (Vector Labs, Peterborough, United Kingdom).

### Planes of section and imaging

Three dimensional reconstructions of the human embryonic and fetal brain, derived from micro computerized tomography scans of whole fetuses, were used to deduce the plane of sectioning and included with the figures presented. The reconstructions were provided by the Human Developmental Biology Resource at https://hdbratlas.org/fetal-stages/8pcw.html, https://hdbratlas.org/fetal-stages/10pcw/, and https://hdbratlas.org/fetal-stages/13pcw.html.

Brightfield images were captured using a using Leica SCN400 Slide Scanner. Fluorescent images were obtained with a Zeiss Axioimager Z2 apotome. Processing of images, which involved adjustment of brightness, color balance and sharpness with minimal removal of artifacts, was achieved using Adobe Photoshop software.

## Results

### Gene expression in the thalamus at 8 PCW

**FIGURE 1:**
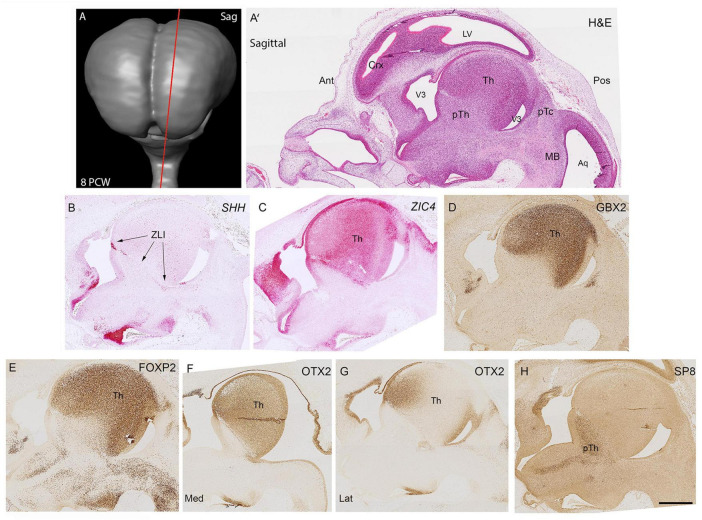
Sagittal sections at 8 PCW. Approximate plane of section and the location of the developing thalamus relative to other structures are shown in **(A,A’)**. The boundary (Zona Limitans, ZLI) between the thalamus (prosomere 2) and prethalamus (prosomere 3) was marked by *SHH* expression **(B)**. *ZIC4* expression marked the entirety of prosomere 2 including thalamus and epithalamic structures **(C)** whereas GBX2 **(D)** and FOXP2 **(E)** immunoreactivity was confined to the thalamus. OTX2 was expressed throughout the medial thalamus **(F)** but in more lateral sections was confined to anterior regions and the ventricular zone **(G)**. SP8 expression, however, served as a marker for the prethalamus **(H)**. Ant, anterior; Aq, aqueduct; Crx, cerebral cortex; H&E, Haemotoxylin and Eosin; Lat, lateral; Med, Medial; MB, midbrain; pTc, pretectum; pTh, prethalamus; Th, thalamus; V3, third ventricle. Scale bar = 1 mm.

The thalamus was clearly recognizable in haemotoxylin and eosin stained sagittal sections as an ovoid structure dorso-anterior to the mesencephalic flexure by 8 post-conceptional weeks (Figure 1A). The precise delineation of the thalamus could be ascertained by examining expression of *SHH* alongside that of 3 transcription factors, *ZIC4*, GBX2 and FOXP2. *SHH* expression marks the Zona limitans (ZLI; the embryonic boundary between p2 (containing the thalamus) and p3 (containing the prethalamus; Kiecker and Lumsden, 2004). It was strongly expressed in cells within the ventricular zone (VZ) at the boundary and in a smaller number of cells along the ZLI (Figure 1B). The alar portion of p2 was characterized by strong expression of *ZIC4* throughout including the dorsal most epithalamus and right up to and including the rostral thalamus close to the ZLI (Figure 1C) although expression was relatively limited in the prethalamus, unlike in the mouse where strong expression in the prethalamus is reported at the equivalent stage of development (E11.5-E12.5) (Li et al., 2018). GBX2 and FOXP2 immunoreactivity was confined to the thalamus and excluded from the epithalamus and showed largely uniform expression throughout (Figures 1D,E) although FOXP2+ cells were more prevalent in the VZ of the thalamus than GBX2+ cells, confirming our previous study (Alzu’bi et al., 2019). OTX2 was also expressed in the thalamus but showed a different pattern of expression, being strongly expressed in both the VZ and post-mitotic zones. This could be predicted from animal models where it has been shown to promote a glutamatergic identity for progeny of thalamic progenitor cells (Puelles et al., 2006). OTX2 was expressed uniformly throughout the thalamus in medial sections, but in lateral sections became confined to regions close to the ventricles (Figures 1F,G). This suggests that OTX2 is expressed by thalamic progenitor cells and more immature, possibly migratory thalamic neurons with expression downregulated as the neurons mature. SP8, a transcription factor associated with GABAergic neurons derived from the ventral telencephalon (Waclaw et al., 2006; Ma et al., 2013; Alzu’bi et al., 2017a) was confined in its expression to the GABAergic prethalamus and not observed in the thalamus in this plane of section (Figure 1H).

**FIGURE 2:**
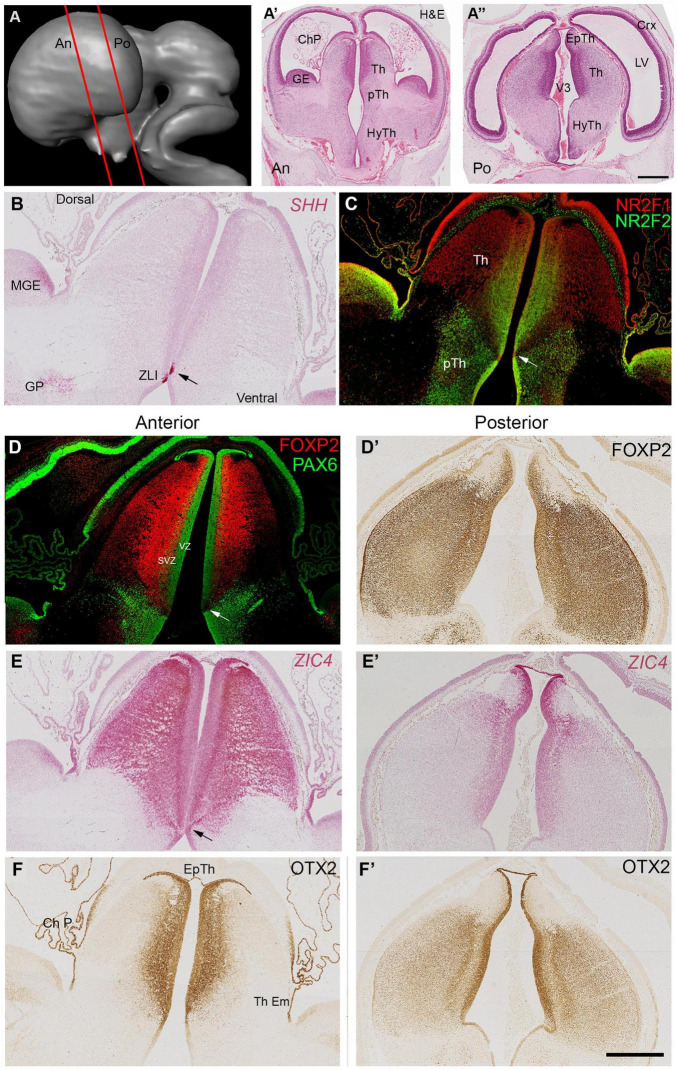
Coronal sections at 8 PCW. Approximate plane of section and the location of the developing thalamus relative to other structures are shown in **(A–A”)**. The ventral boundary of the thalamus (Th) marked by expression of SHH in a small group of cells located in the ventricular zone around the 3rd ventricle (ZLI, arrows, **B**). NR2F1 predominantly expressed dorsal of the ZLI, whereas NR2F2 predominantly a marker for the prethalamus (pTh) although there was also clear expression in ventromedial thalamus **(C)**. PAX6 expression in both thalamus and epithalamus was strong in the ventricular zone (VZ) **(D)**. Strength of PAX6 expression decreased in a dorsal to ventral gradient, disappearing altogether at the ZLI (arrow) but reappearing strongly in the prethalamic VZ and in post-mitotic cells in more lateral prethalamic locations. It was also present in some cells of the thalamic subventricular zone (SVZ). FOXP2 expressed predominantly in thalamic post-mitotic neurons. More anteriorly **(D)** expressed throughout the thalamus, more strongly near the midline and excluded from the epithalamus. More posteriorly **(D’)** expression strong throughout the thalamus. Conversely *ZIC4* expression **(E,E’)** was strong throughout the anterior thalamus, but weaker and confined dorsally and medially posteriorly. *ZIC4* also detected in the epithalamus and the VZ of prosomere 2. OTX2 also expressed in the epithalamus (Ep Th) and the VZ, as well as the choroid plexus (ChP) and thalamic eminence (Th Em). Like FOXP2, generally more strongly expressed posteriorly **(F,F’)** and medially. Scale bar = 1 mm.

**FIGURE 3:**
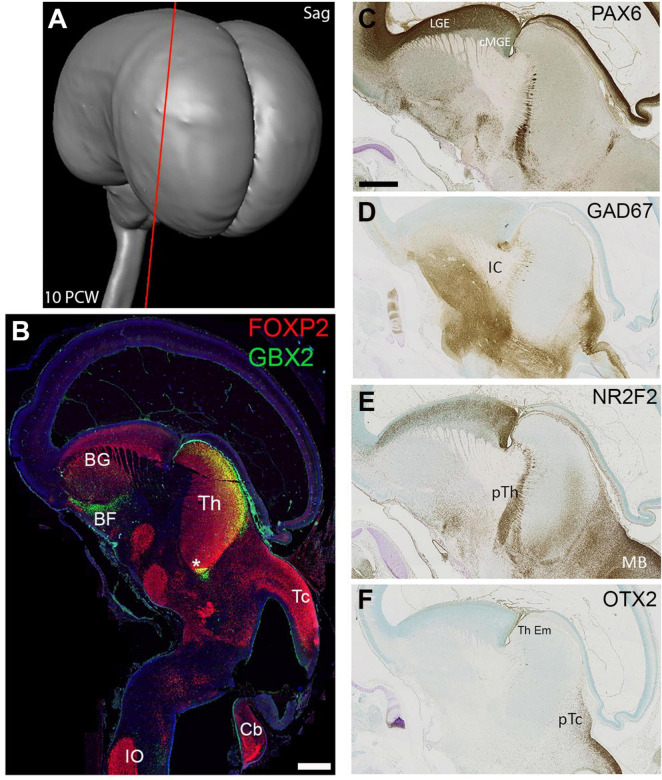
Sagittal sections at 10 PCW. Approximate plane of section shown in **(A)**. By 10 PCW, FOXP2, and GBX2 both strongly expressed in the thalamus; FOXP2 throughout, GBX2 confined to more dorsal posterior locations and areas close to the third ventricle (asterisk**, B**). FOXP2 also expressed at multiple other sites in the basal ganglia, hypothalamus, subthalamus, midbrain and hindbrain. GBX2 expressed by neurons of the basal forebrain and basal ganglia (**B**, Chen et al., 2010). PAX6 expression was excluded from the mantle of the thalamus but expressed by cells marking the boundary between thalamus and prethalamus **(C)**. GAD67 immunoreactivity was excluded from the thalamus, but strongly expressed by cell bodies and fibres in the hypothalamus, subthalamus, pretectum and midbrain **(D)**. NR2F2 also expressed in the prethalamus as well as pretectum and midbrain, but, unlike PAX6, clearly expressed by a proportion of cells in the ventral posterior thalamus **(E)**. OTX2 expression not observed in the mantle of the thalamus but seen in the adjoining pretectum and thalamic eminence **(F)**. Section **(B)** counterstained with DAPI (blue). BF, basal forebrain; BG, basal ganglia; Cb, cerebellum, cMGE, caudal medial ganglionic eminence; IC, internal capsule; IO, inferior olive; LGE, lateral ganglionic eminence; pTc, pretectum; pTh, prethalamus; Tc, tectum; Th, thalamus; Th Em, thalamic eminence. Scale bars = 1 mm.

Coronal sections (locations shown in Figures 2A–A”) confirmed these observations, *SHH* expression was clearly observed in a small group of cells in the thalamic VZ just dorsal to the hypothalamic sulcus (Figure 2B). Gene expression in this small region differed from the VZ both dorsal and ventral to it, showing absence of immunoreactivity for NR2F2 and PAX6 (Figures 2C,D). NR2F1 and *ZIC4* were expressed throughout prosomere 2 including the VZ and the epithalamus (Figures 2C,E) whereas FOXP2 immunoreactivity was confined to the thalamus except for the VZ and excluded from the epithalamus. Expression of FOXP2 was also relatively weak in more lateral regions of the thalamus, compared to *ZIC4* and NR2F1. PAX6 was expressed throughout the VZ of p2 (Figure 2D). However, there was a ventral low to dorsal high gradient of expression. Some PAX6+ cells were present in the subventricular zone of the thalamus (SVZ). Ventral to the ZLI, PAX6 immunoreactivity was high in the VZ of the prethalamus, and PAX6 expressing post-mitotic cells were present throughout the prethalamus, medially to laterally (Figure 2D). NR2F2 was also expressed strongly in the prethalamus, both ventricular and post-mitotic layers (Figure 3B) as well as in the thalamic VZ in a ventral high to dorsal low gradient, that is, the opposite of PAX6. It was also expressed in post-mitotic cells in more medial locations in ventral parts of the thalamus.

Figures 2D–F’ compare more anterior and posterior sections of thalamus for expression of three transcription factors that are key to thalamic development. FOXP2 expression was seen throughout the thalamus, but was absent from the epithalamus and weakly expressed in more lateral regions of the anterior thalamus (Figures 2D,D’). *ZIC4*, on the other hand, was strongly and uniformly expressed throughout the anterior thalamus and epithalamus, but showed weaker expression posteriorly, confined to the epithalamus and medial and dorsal thalamic areas close by Figures 2E,E’. Localization of OTX2 immunoreactivity in prosomere 2 was more similar to the FOXP2 expression pattern, except that is was expressed in the epithalamus, strongly expressed in the ventricular zone, and was more weakly expressed laterally, especially anteriorly (Figures 2F,F’).

As predicted from animal experiments (Puelles et al., 2013) expression patterns of transcription factors and other molecules clearly delineate p2 from p1 and p3, with p2 and p3 being separated by the SHH positive ZLI. The thalamus can also be distinguished from the epithalamus at this stage, with only *ZIC4*, OTX2 and PAX6 being expressed in the epithalamus. The thalamic VZ is divided into zones, with stronger PAX6 expression dorsally and stronger NR2F2 expression close to the ZLI. In post-mitotic zones, *ZIC4* is expressed in a high anterior to low posterior gradient, whereas the gradient of expression of FOXP2 and OTX2 opposes this.

### Gene expression at 10 PCW

In sagittal sections at 10 PCW (Figure 3A) the thalamus had become more elongated dorsally to ventrally, compared to 8 PCW and appeared bean shaped (Figure 3B). Its full extent was clearly delineated by FOXP2 immunoreactivity, but by this stage GBX2 expression was confined either to dorsal posterior regions of the thalamus, or close to the 3rd Ventricle (Figure 3B). FOXP2 was also expressed in numerous other brain regions nearby, while GBX2 was also expressed by cells of the basal forebrain and basal ganglia, presumably precursors of cholinergic interneurons of the basal ganglia, and non-cholinergic neurons of the basal forebrain as observed in mouse (Chen et al., 2010). Other gene expression patterns revealed the boundaries of the thalamus. PAX6 and NR2F2 were strongly expressed in the prethalamus (Figures 3C,D) although weak NR2F2 immunoreactivity was also observed in the ventral posterior thalamus. GAD67 immunoreactive neurons and axons were excluded from the thalamus but were abundant in the prethalamus, subthalamus, hypothalamus, basal ganglia and pretectum (Figure 3E). Similarly, OTX2 expression marked the pretectum and thalamic eminence, but could not be seen in the thalamus in these sections (Figure 3F).

**FIGURE 4:**
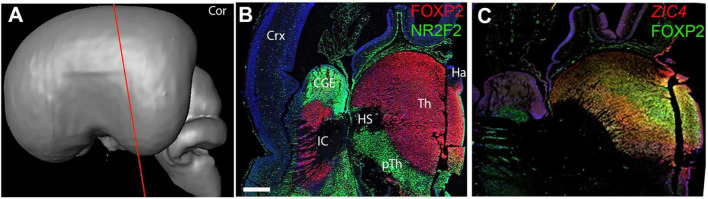
Coronal sections at 10 PCW. **(A)** Approximate plane of section. **(B)** Largely complementary expression patterns of FOXP2 and NR2F2 observed with FOXP2 marking the post-mitotic zones of the thalamus and NR2F2 immunoreactivity marking the prethalamus and thalamic VZ. *ZIC4* and FOXP2 expression overlap although ZIC4 expression more uniform throughout prosomere 2 including the habenula, FOXP2 more strongly expressed medially **(C)**. Sections **(B,C)** counterstained with DAPI (blue). CGE, caudal ganglionic eminence; Ha, habenula; HS, Hemispheric stalk: IC, internal capsule; pTh, prethalamus; Tc, tectum; Th, thalamus. Scale bar = 1 mm. Panel **(C)** is adapted from Alzu’bi et al. (2022) with permission of the publisher.

Coronal sections (Figure 4A) confirmed these findings. Figure 4B clearly shows FOXP2 immunoreactivity throughout the extent of the thalamus but excluded from the habenula (derived from the epithalamus) with NR2F2 strongly expressed in the prethalamus, but also in the thalamic VZ. As at 8 PCW, at a relatively anterior level, *ZIC4* was expressed throughout both thalamus and habenula, in mitotic and post-mitotic cells. Co-staining with FOXP2 revealed that *ZIC4* showed a relatively homogenous expression, whereas FOXP2 showed relatively stronger expression in mediodorsal regions (Figure 4C).

**FIGURE 5:**
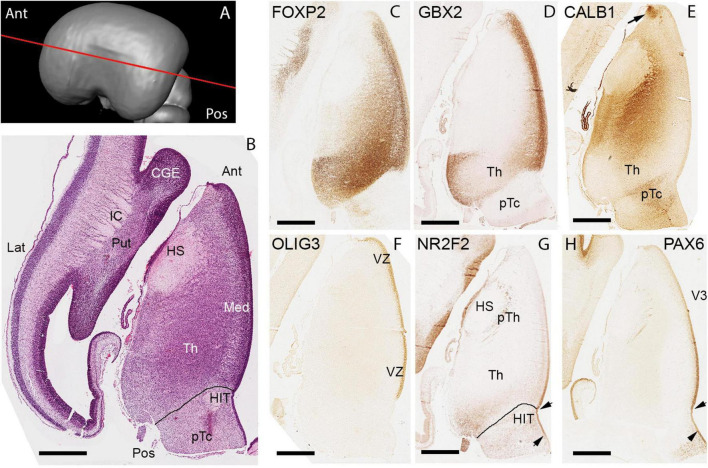
Horizontal sections at 10 PCW. **(A)** Approximate plane of sectioning. **(B)** H&E stained section with anatomical features annotated. Horizontal sections revealed more clearly regional differences in gene expression throughout the thalamus by this developmental stage. FOXP2 present throughout thalamus but most strongly expressed in posterior regions **(C)**. GBX2 showed strong expression medially but also in posterio-lateral locations **(D)**. Calbindin (CALB1) strongly expressed in thalamic areas where GBX2 was not, including the epithalamus (arrow, **E)**. OLIG3 expressed throughout the VZ of the thalamus and epithalamus **(F)**, but both PAX6 **(G)** and NR2F2 **(H)** exhibited a gradient of expression within the VZ with expression stronger posteriorly. NR2F2 also expressed by some post-mitotic cells of posterior thalamus. The prethalamic area around the hemispheric stalk (HS) characterized by presence of PAX6+ and NR2F2+ cells. Lines in **(B,G)** mark thalamic/pretectal boundary. Arrowheads in **(G,H)** mark extent of the PAX6 low expression in pretectal VZ. CGE, caudal ganglionic eminence; HIT, Habenulo interpeduncular tract; IC, internal capsule; pTc, pretectum; Put, putamen; Ant, anterior; Lat, lateral; Med, medial; Pos, Posterior. Scale bars: 1 mm in **(B)**, 500 μm in **(C–H)**.

Horizontal sections (Figures 5A,B) further confirm and expand upon these findings. FOXP2 expression delineated the extent of the thalamus whereas GBX2 immunoreactivity was confined to medial regions just lateral of the VZ but also to posterior-lateral parts of the thalamus (Figures 5C,D). Interestingly, in regions where GBX2 expression was reduced, calbindin (CALB1) immunoreactivity was observed (Figure 5E). In the ventricular zone, OLIG3 expression confirmed the extent of p2 as observed in mouse (Figure 5F; Takebayashi et al., 2002; Vue et al., 2007) and suggested by human ScRNAseq studies (Kim et al., 2023). NR2F2 was also expressed in the thalamic VZ in a gradient with strong expression posteriorly and weak anteriorly and was also expressed by cells in the posterior-lateral thalamus, but not in exactly the same locations as where GBX2 immunoreactivity was observed (Figures 5D,G). PAX6 was also expressed in a gradient in the VZ with strong expression posteriorly and weak anteriorly (Figure 5H). PAX6 expression was reduced in the VZ just at the boundary with the pretectum, but was expressed in the VZ and some post-mitotic cells posterior of the boundary.

In summary, the boundaries of the thalamus can be clearly delineated by the expression patterns of various mRNA and proteins. The VZ of the thalamus continues to show some evidence of a protomap, with gradients of expression of both PAX6 and NR2F2 observed. Although defined thalamic nuclei were not identifiable at this stage, post-mitotic cells also show evidence of regionalized gene expression by 10 PCW, with GBX2 expression, in particular, becoming confined to medial and posterior locations and CALB1 immunoreactivity occupying complementary lateral locations. FOXP2 expression, while ubiquitous, was stronger in posterior and medial locations.

### Emergence of thalamic nuclei at 14/15 PCW

**FIGURE 6:**
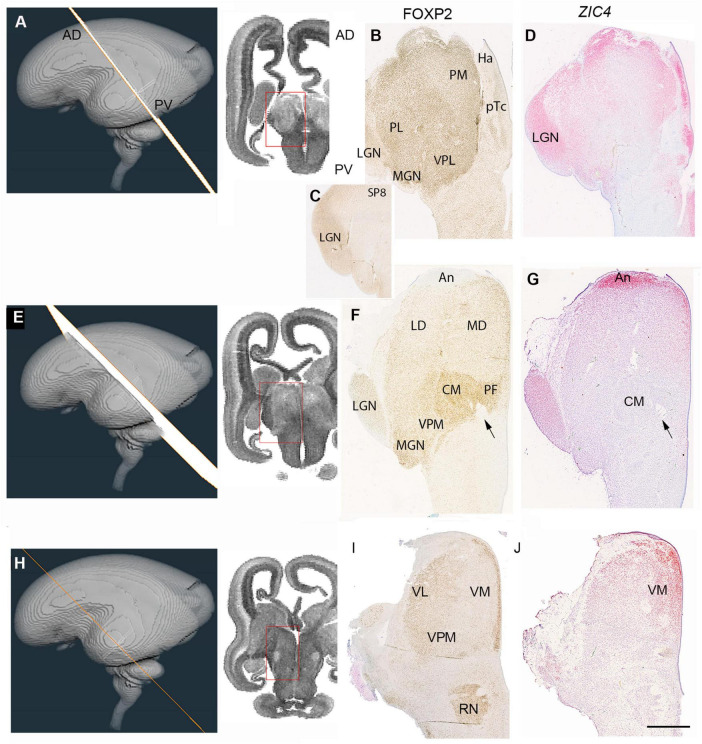
Emergence of the thalamic nuclei 14 PCW- FOXP2 and *ZIC4* expression. **(A)** The plane of sectioning for the anterior dorsalmost set of sections mostly containing pulvinar, medial geniculate nucleus (MGN) and lateral geniculate nucleus (LGN). **(B)** Strong FOXP2 immunoreactivity across the thalamus with a slight increasing gradient from medial to lateral. SP8 strongly expressed in the LGN **(C)**. *ZIC4* expression throughout the thalamus and habenula (Ha) with strongest expression anteriorly, medially and in the LGN **(D)**. **(E)** The plane of section for a set of sections from the middle of the thalamus. **(F)** FOXP2 differentially expressed in discrete thalamic nuclei, with highest expression in the centromedial nucleus (CM) and associated parafascicular nucleus (pF) close to the habenulo peduncular tract (arrow) and weakest in anterior thalamus (An). *ZIC4* strongly expressed anteriorly and in the LGN **(G)**. **(H)** The plane of section for the posterior ventralmost set of sections. FOXP2 expression strong in the paraventricular regions and moderately strong laterally although excluded from the thalamic reticular formation **(I)**. *ZIC4* showed strongest expression in anterior and medial regions of the thalamus **(J)**. AD, anterior dorsal; LD, laterodorsal; MD, mediodorsal; PV, posterior ventral; VL, ventrolateral nucleus; LP, lateral pulvinar; VPM, ventral posteriomedial nucleus. Scale bar = 1 mm.

**FIGURE 7:**
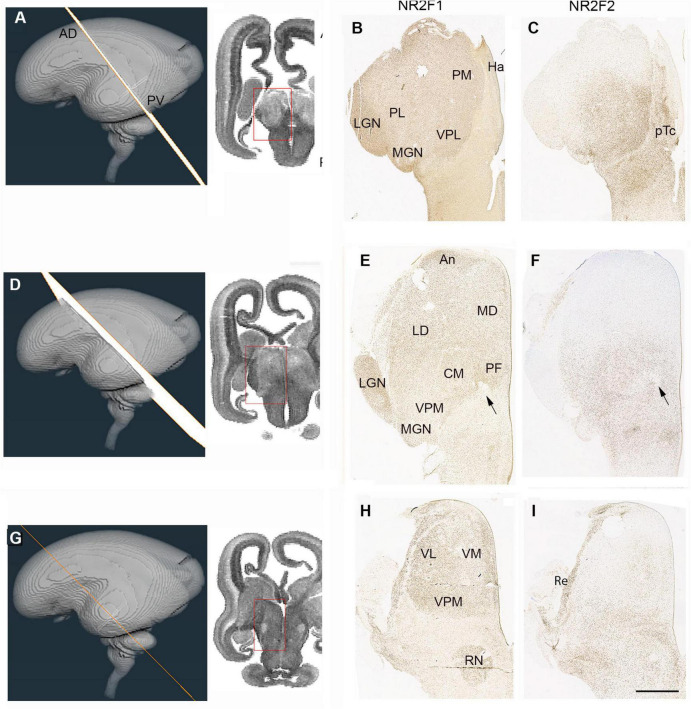
Emergence of the thalamic nuclei 14 PCW- NR2F1 and NR2F2 expression. **(A)** The plane of sectioning for the anterior dorsalmost set of sections mostly containing pulvinar, medial geniculate nucleus (MGN) and lateral geniculate nucleus (LGN). **(B)** Demonstrates strong NR2F1 expression throughout the thalamus at this level, strongest immunoreactivity in the LGN. NR2F2, however, showed strongest expression in the pretectum (pTc). In the thalamus was confined to more ventral and medial regions corresponding to ventral posteriolateral nucleus (VPL) and inferior pulvinar **(C)**. **(D)** The plane of section for a set of sections from the middle of the thalamus. NR2F1 immunoreactivity was high across the thalamus, strongest anteriorly and in the LGN **(E)**. NR2F2 expression confined to the posterior half of the thalamus, except for the LGN **(F)**. Arrows indicate habenulo-peduncular tract. **(G)** The plane of section for the posterior ventralmost set of sections. NR2F1 strongly expressed throughout the thalamus and in the red nucleus (RN; **H**). NR2F2 strongly expressed in the thalamic reticular formation (Re) and weakly expressed in medial and posterior thalamus **(I)**. AD, anterior dorsal; LD, laterodorsal; Ha, habenula; MD, mediodorsal; PV, posterior ventral; PL, lateral pulvinar; VL, ventrolateral nucleus; VM, ventromedial nucleus; VPM, ventral posteriomedial nucleus. Scale bar = 1 mm.

**FIGURE 8:**
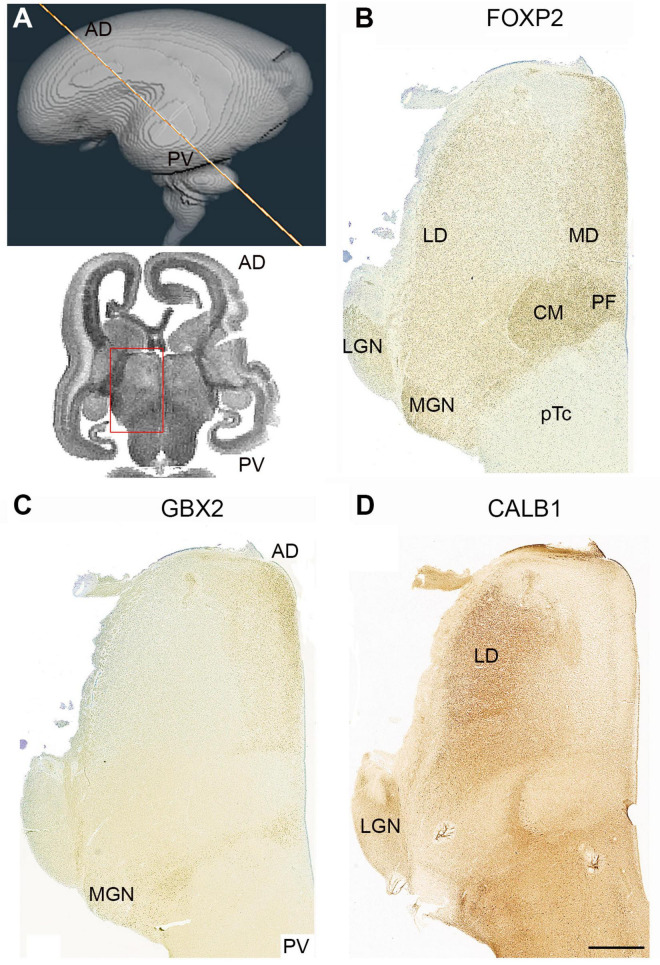
Complementary expression patterns in the thalamus at 14 PCW. **(A)** The approximate plane of sectioning. FOXP2 **(B)** GBX2 **(C)** and calbindin (CALB1; **D**) showed complementary expression in some thalamic nuclei and co-expression in others. The medial dorsal nucleus (MD) and the medial geniculate nucleus (MGN) exhibited co-expression of FOXP2 and GBX2, the lateral dorsal nucleus (LD) and lateral geniculate nucleus (LGN) showed co-expression of FOXP2 and CALB1. The centromedian nucleus (CM) and parafascicular complex (pF), however, only expressed FOXP2. AD, anterior dorsal; PV posterior ventral; 3V, third ventricle. Scale bar = 1 mm.

**FIGURE 9:**
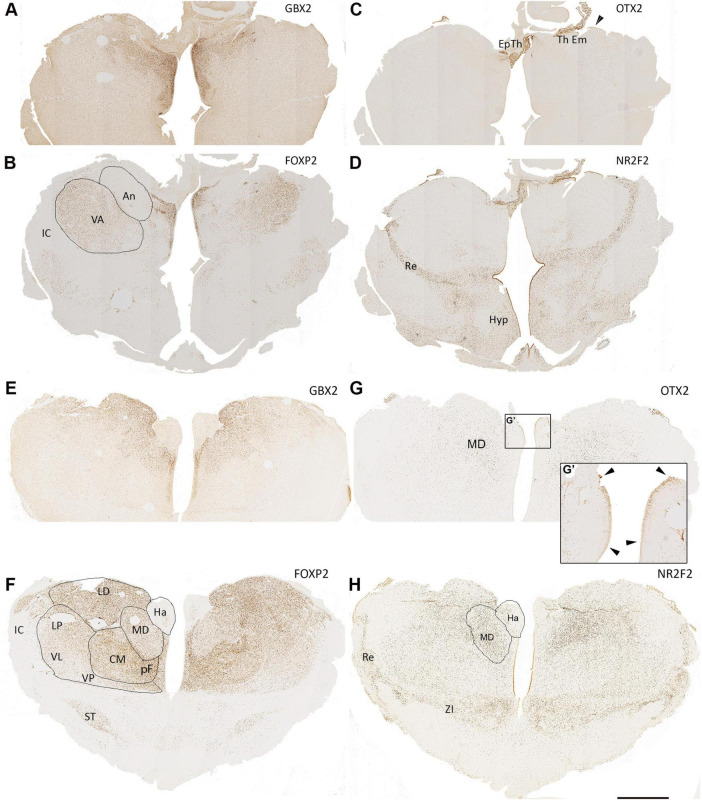
15 PCW coronal sections. Anterior sections of the diencephalon, **(A**,**B)**, GBX2 and FOXP2 show partially complementary patterns of expression with FOXP2 expressed in the ventral anterior nucleus (VA) and GBX2 expressed in the anterior nucleus (An) with co-expression in medial structures. Both excluded from the epithalamus (Ep Th) and thalamic eminence (Th Em) which strongly express OTX2 **(C)**. NR2F2 also strongly expressed in these structures **(D)** as well as in the reticular formation (Re) and parts of the hypothalamus (Hyp). Also, some NR2F2+ cells can be seen within medial ventral thalamus as well as in the thalamic ventricular zone in a weakening ventral to dorsal gradient. In sections from the middle of thalamus, **(E,F)**, GBX2 strongly expressed in laterodorsal (LD) and mediodorsal (MD) regions, whereas FOXP2 expression is reduced in the MD but highly expressed in the more ventral centromedian nucleus (CM) and parafascicular complex (pF). Both transcription factors are excluded from the habenula (Ha), however, OTX2 is strongly expressed in the Ha ventricular zone (**G**,**G’**, arrowheads). OTX2 also expressed by scattered cells in more medial and dorsal thalamic regions **(G)**. NR2F2 expression marked the reticular formation and zona incerta (ZI) but also seen in cells throughout the thalamus, particularly in the MD **(H)**. IC, internal capsule; ST, subthalamic nucleus. Scale bar = 1 mm.

**FIGURE 10:**
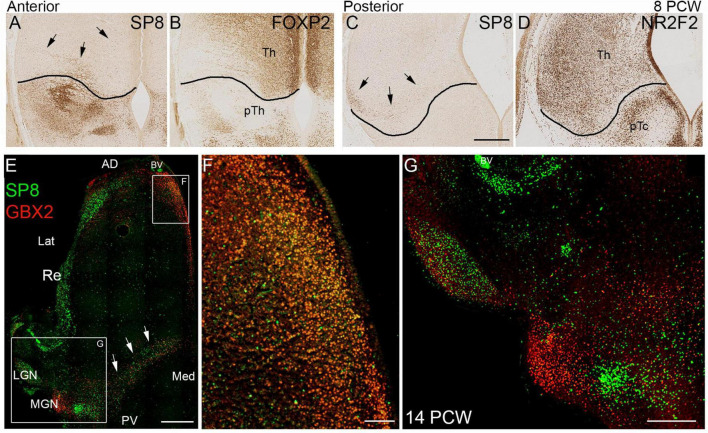
SP8 expression in the thalamus. **(A)** SP8 immunoreactivity in a coronal section of anterior thalamus. The black line represents the boundary between thalamus (Th) and prethalamus (pTh) derived from an adjacent section immunostained for FOXP2 (**B;** see also Figure 3). Black arrows indicate SP8 positive cells clearly located within the thalamus. Similarly **C** illustrates a small group of SP8 positive cells in a ventrolateral location in the thalamus in a posterior coronal section, close to the boundary with the pretectum (pTc). Boundary derived from an adjacent NR2F2 immunostained section (**D**; see also Figure 3). **(E)** Illustrates double immunofluorescent staining for SP8 and GBX2 at 14 PCW. Many cells double labeled for SP8 and GBX in anterior medial location **(E,F)**. SP8 singly expressed in the reticular formation (Re) lateral geniculate nucleus (LGN) and a group of cells just ventromedial to the GBX2+ medial geniculate nucleus (MGN; **E,G**). GBX2+ and SP8+ cells intermingled along posterior border of the thalamus, relatively few were double labeled (arrows; **E**). AD, anterior dorsal; BV, blood vessel; Lat, lateral; Med, medial; PV posterior ventral. Scale bars = 200 μm **(A–D,F)**; 1 mm **(E)**; 500 μm **(G)**.

We considered two series of sections through the thalamus, one extensive set from a 14 PCW specimen stained for multiple mRNAs and proteins and cut in a plane intermediate between horizontal and coronal, the exact plane deduced by comparing the histological sections with virtual sections from a 3-D MRI image of the fetal brain supplied by the Human Developmental Biology resource (Figures 6A,E,H, 7A,D,G, 8A)^3^ and a smaller set of coronal sections from a 15 PCW immunostained for a limited number of proteins (Figure 9). By 14 PCW it was clear that the thalamus was becoming divided into discrete regions identifiable by patterns of gene expression and containing relatively cell dense and cell poor regions (Figures 6, 7). FOXP2 localization provided the most striking visualization of putative thalamic nuclei (Figures 6B,F,I, 10B). In more dorsal and anterior sections (Figure 6A) the extent of the pulvinar complex was delineated by moderate to strong FOXP2 expression, with expression stronger in the lateral and inferior pulvinar regions compared to medial pulvinar and the lateral geniculate nucleus (LGN), which we were able to observe as a discrete nuclei by this developmental stage (Figure 6B). The LGN occupied a more dorsal location lateral to the main body of thalamus than where it is found in the adult thalamus. As has been previously described, the late developing pulvinar gradually displaces the LGN in a latero-ventral direction (Hitchcock and Hickey, 1980). The LGN had not adopted a laminar structure by 14 PCW also confirming what was reported previously (Hitchcock and Hickey, 1980). The LGN which also showed strong expression of SP8 (Figure 6C). *ZIC4* was no longer expressed in all of the anterior part of p2 but was still strongly expressed in the putative habenula and the antero-dorsal edge of the thalamus at this level, as well as some medial parts of the pretectum. It was also strongly expressed in the LGN (Figure 6D).

In sections located more posteriorly and ventrally (Figures 6E–G) distinct boundaries between thalamic nuclei, as well as between thalamus and pretectum, were observed. FOXP2 expression was very strong in the putative centromedian nucleus (CM) and parafascicular complex (pF). It was also moderately expressed close to the midline and in the MGN, but immunoreactivity was less in lateral parts of the thalamus (Figure 6F). Strong *ZIC4* expression was confined to the anterior thalamus and LGN, where FOXP2 expression was low, but was absent from the CM-PF where FOXP2 expression was high (Figures 6F,G). The most posterior and ventral set of sections (Figures 6H–J) offered a different perspective. Here, the paraventricular complex stained strongly for FOXP2, otherwise there seemed to be a gradient of increasing FOXP2 from medial to lateral, distinguishing a putative ventromedial nucleus from more lateral structures (Figure 6I). *ZIC4* showed more widespread expression at this level but was strongest in the anterodorsomedial parts of the section (Figure 6J).

NR2F1 and NR2F2 are involved in the patterning of the human telencephalon and show partly complementary, partly overlapping patterns of expression during early fetal development (Alzu’bi et al., 2017a). In anterior and dorsal sections (Figure 7A) NR2F1 showed high expression in the diencephalon which was largely exclusive to the thalamus but with relatively higher expression in the LGN (Figure 7B) confirming a previous scRNAseq/spatial transcriptomic study in mid-gestation human brain (Kim et al., 2023). NR2F2, showed a partly complementary pattern, with strong immunoreactivity in the pretectum, but it was also expressed in the medio-posterior parts of the thalamus (Figure 6D), as was observed at 10 PCW (Figure 5G). In sections located more posteriorly and ventrally (Figures 7D–F) again, NR2F1 expression was strong in the thalamus and weak in the pretectum (Figure 7E). Expression was particularly marked in the LGN and in an anterior region that lacked FOXP2 immunoreactivity but showed strong expression of *ZIC4* (Figures 6F,G). NR2F2 expression was strong in the pretectum and extended into the most ventroposterior half of the thalamus, covering the CM-pF complex and also areas lateral to it, but excluding the LGN (Figure 7F). In the most posterior and ventral set of sections NR2F1 showed a similar pattern of expression as seen more dorsally but was also detected in the thalamic reticular formation, which predominantly originates from p3 (Puelles et al., 2013; Puelles and Rubenstein, 2003) and forms a lateral boundary to the thalamus (Figure 7H). The reticular formation was likewise strongly immunoreactive for NR2F2, which was also expressed in the pretectum and midbrain but extended expression into the dorsomedial and ventrolateral regions of the thalamus as well (Figure 7I).

The observation of SP8 expression in the LGN at 14PCW was a surprising finding so we then further examined SP8 immunoreactivity at 8 PCW in comparison to thalamic markers (Figures 10A–D) and found evidence of low levels of expression within the thalamus, close to the border with the prethalamus anteriorly, where SP8 was expressed strongly (Figure 10A) and pretectum posteriorly, where SP8 was absent (Figure 10C). A defined group of SP8+ cells could be seen ventrolaterally in posterior sections (Figure 10C). We also examined several levels at 14 PCW and found it to be a reliable marker for the LGN. At more ventral levels, it was also expressed along the posterior ventral boundary and in medial anterior regions. Both areas expressed GBX2 and in a double labeled preparation we found, in anterior regions, SP8 was co-expressed with GBX2 in a majority of cells (Figures 10E,F). However, in posterior ventral regions, SP8 and GBX2 immunoreactivity were expressed in the same region but mostly in different cells (Figures 10E,F). The medial geniculate nucleus (MGN) only expressed GBX2, but there was a small grouping of cells just medial and posterior to the MGN that strongly expressed SP8 exclusively (Figure 10F). This was in addition to strong expression in the thalamic reticular nucleus (Figure 10E). This is the first time, to our knowledge, that SP8 expression has been described in the developing thalamus.

GBX2 expression was weak at this stage of development and not always easy to detect, but in Figures 8C, 10E–G, it can be seen to be expressed anteriorly and medially, and in restricted locations posteriorally, as described at 10 PCW (Figure 5D). It also forms a complementary pattern of expression with CALB1 (Figures 8C,D) as described at 10 PCW, except that neither marker is expressed in the CM and the medial geniculate nucleus (MGN) where there is strong immunoreactivity for FOXP2. However, FOXP2 and GBX2 were both expressed in the putative medial dorsal nucleus (Figures 8B,C).

**FIGURE 11:**
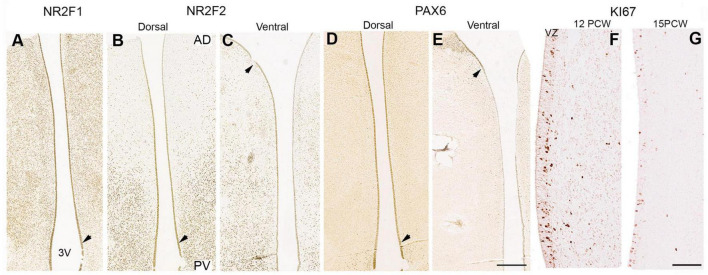
Transcription factor expression and cell division in the ventricular zone. In the ventricular zone (VZ) at 14 PCW a thin layer of cells retained expression patterns seen at earlier stages when neurogenesis was ongoing. NR2F1 expression found throughout thalamic VZ **(A)**. NR2F2 expression decreased from posterior to anterior, especially in more dorsal sections **(B,C)**. PAX6 expression was stronger dorsally than ventrally, posteriorly than anteriorly, and strongest in the pretectal (**D**; unlike what was observed at 10 PCW; Figure 5H) and prethalamic VZ, where positive cells can also be seen laterally **(E)**. Downward pointing arrowheads mark boundary between thalamus and pretectum, upward arrowhead marks boundary between thalamus and prethalamus/reticular formation. **(F,G)** Higher magnification images of thalamic VZ at 12 and 15 PCW showing expression of cell division marker KI67 predominantly in the VZ but also scattered through post-mitotic zones. AD, anterior dorsal; PV posterior ventral; 3V, third ventricle. Scale bars = 1 mm (**A–E)**; 100 μm **(H,I)**.

Cells lining the third ventricle remained positive for transcription factors expressed earlier in development. NR2F1 expression was homogenous throughout the thalamus (Figure 11A), however, NR2F2 expression was high close to the midbrain boundary but reduced in cells in more anterior locations (Figures 11B,C) whereas PAX6 showed higher expression dorsally (Figure 11D) and low expression ventrally and close to the boundary with p3 (arrow; Figure 11E) similar to the pattern observed at 10 PCW (Figure 5). Employing tritiated thymidine labeling, neurogenesis in the human thalamus is reported to be complete by 15 PCW (Rakic and Sidman, 1968). We found immunoreactivity for the cell division marker KI67 in the ventricular zone of the thalamus at both 12 and 15 PCW (Figures 11F,G) although this may be an indicator of gliogenesis as well as neurogenesis. Therefore, thalamic neurogenesis may be fairly protracted, and cell identity may be determined by a protomap until well into the second trimester.

Coronal sections of 15 PCW thalamus offer a different perspective, giving more access to anterior and ventral regions. In the most anterior sections, GBX2 expression was confined to the more medial regions of the thalamus, except for in the most dorsal regions where it extends laterally into the putative anterior nucleus (Figure 9A). FOXP2, on the other hand, was confirmed to be co-expressed with GBX2 medially, was not expressed in the anterior nucleus, but did show strong expression in the putative ventral anterior nucleus, more ventrally and laterally (Figure 9B). OTX2 was strongly expressed in the ventricular zone of both the epithalamus, choroid plexus and thalamic eminence, but exhibited no expression in the thalamus itself (Figure 9C). NR2F2 was also strongly expressed in the VZ of the thalamic eminence and epithalamus, and also in the reticular formation and regions of the hypothalamus, but similarly was not expressed in the thalamus at this anterior location (Figure 9D).

For sections from more towards the middle of the thalamus, GBX2 was most strongly expressed medially in the pF and MD (Figure 9E). Expression was completely absent from the putative habenula, which was also devoid of FOXP2 immunoreactivity (Figure 9F), but showed OTX2 immunoreactivity throughout its ventricular surface, unlike the thalamus (Figures 9G,G’). FOXP2 was expressed throughout the thalamus, but expression was strongest in the CM, and weakest in lateral regions. It was also expressed in the subthalamic nucleus (Figure 9F). Strong NR2F2 expression was observed the reticular formation and zona incerta between the thalamus and subthalamus (Figure 9H). However, it was also expressed throughout the thalamus except for the most dorso-lateral regions confirming observations made at 14 PCW. Interestingly, OTX2 immunoreactivity within the thalamus, although weaker than NR2F2, was also most highly expressed in post-mitotic cells of the MD (Figure 9G).

### Invasion of the thalamus by GABAergic neurons

**FIGURE 12:**
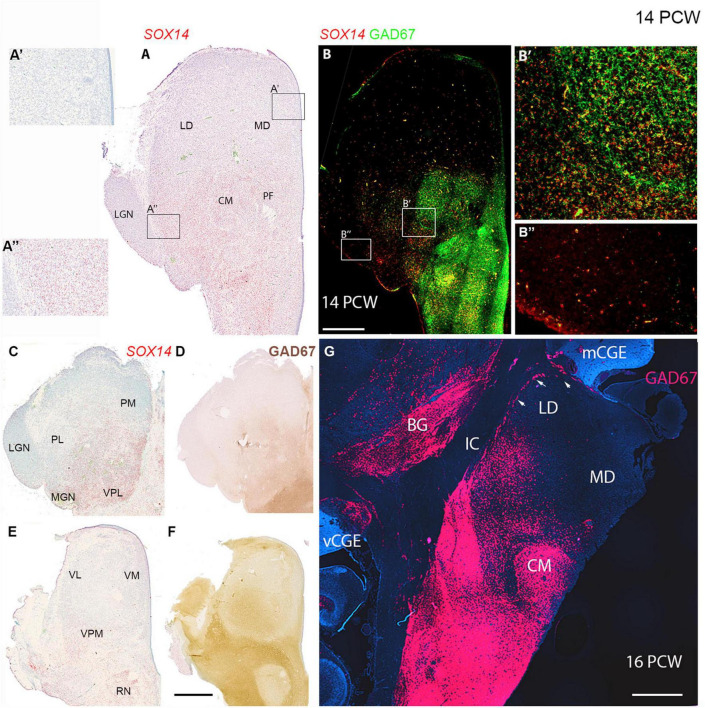
Thalamic GABAergic neurons and their precursors. **(A)** Transcription factor *SOX14* expressed in midbrain, pretectum and in more posterior parts of the thalamus at a mid-thalamic level. Expression almost entirely absent from anterior parts of the section **(A’)**. Expression stronger in lateral posterior thalamus, but weak in the adjacent lateral geniculate nucleus (LGN) **(A”)**. **(B)** Co-expression (yellow) of GAD67 and *SOX14* in the midbrain, pretectum and the thalamic centromedial nucleus (CM) and parafascicular complex (PF) including at the cellular level **(B’)**. In more lateral posterior regions, *SOX14* expressed singularly **(B’)** and at low levels in the LGN **(B”)**. In more dorsal sections **(C,D)** *SOX14* and GAD67 expression was confined to the same medial and posterior parts of the thalamus corresponding to lateral and inferior pulvinar and posterior parts of medial pulvinar. In more ventral sections **(E)** *SOX14* expression relatively weak and largely confined to lateral margins. It did not correspond particularly strongly with GAD67 immunoreactivity at this level **(F)**. **(G)** GAD67 expression in a coronal section of thalamus at 16 PCW, 2 weeks later than panels **(A–F)**. Expression strong ventral to the thalamus and also in the thalamic CM but decreased dorsally and was absent from the mediodorsal nucleus (MD). A line of GAD67+ cells (arrows) found at the lateral margin of the laterodorsal nucleus (LD) which may represent the reticular nucleus. It is contiguous with MGE-like CGE (mCGE) and may include neurons migrating away from this proliferative zone. BG, basal ganglia; IC, internal capsule; vCGE, ventral caudal ganglionic eminence. Scale bars = 1 mm.

At 10 PCW no GAD67 immunoreactive neuronal cell bodies were observed in the thalamus (Figure 3D) in agreement with a recent ScRNAseq study (Kim et al., 2023). However, by 14 PCW three potential markers for GABAergic neurons or their precursors, NR2F2, *SOX14*, and GAD67, were all generally expressed in the more posterior parts of the thalamus. Expression of all four was greatest in the pretectum and midbrain, and appeared in a gradient across the thalamus, high at the thalamo-pretectal border and declining towards dorsal and anterior thalamus (Figures 7C,F,I, 10D, 12). These patterns of expression generally overlapped, suggesting a migration of cells from pretectum and midbrain into posterior regions of the thalamus.

GAD67 exhibited particularly striking expression in the CM-pF, as has been previously been shown, at 16 PCW (Alhesain et al., 2023; Figures 12B,D,F,G) where it showed clear co-expression with *SOX14* in individual cells (Figure 12B’). However, GAD67 was not expressed just lateral to these nuclei where NR2F2 and SOX14 expression was observed (Figure 12B”). NR2F2 expression was seen in the FOXP2 negative reticular formation, along with lower levels of GAD67 and SP8 immunoreactivity. However, no SP8+ positive neurons were present in the thalamus, except in the dorsal LGN as described above.

We conclude that, between 10 and 14 PCW, the thalamus is partially invaded by *SOX14+* and possibly NR2F2+ cells of pretectum/midbrain origin, however, certain nuclei, for instance the medial dorsal nucleus and medial pulvinar, remain devoid of these cells even by 16 PCW, suggesting migration has stopped. However, in some locations, maturation of these cells is quicker than in others. For instance the *SOX14+* cells of the CM express GAD67 at this stage, whereas those of the LGN do not.

### Differential expression of the NND susceptibility gene *CNTNAP2* in the developing thalamus

**FIGURE 13:**
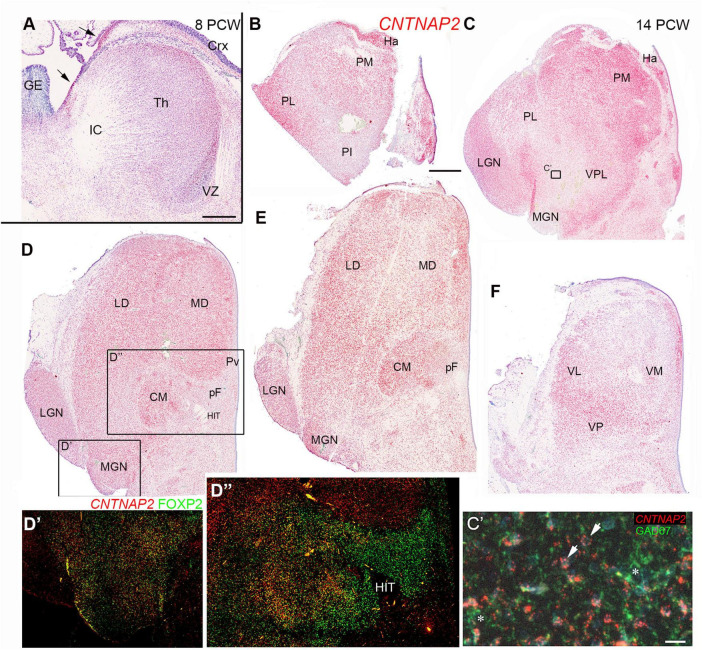
C*NTNAP2* expression in the developing thalamus. **(A)** Widespread expression of *CNTNAP2* in post-mitotic cells of the thalamus (th) and (Crx), strong expression in the thalamic eminence and cortical hem (arrows) but low expression in the thalamic ventricular zone (VZ) and proliferative regions of cortex and ganglionic eminence (GE). **(B–F)** A series of sections at 14 PCW from anterior dorsal to posterior ventral thalamus at 14 PCW. **(B,C)** *CNTNAP2* strongly expressed in habenula (Ha) and medial and lateral pulvinar (PM and PL) but less in the inferior pulvinar (PI). Expression moderate in the ventroposterolateral nucleus (VPL) and lateral geniculate nucleus (LGN). Low expression in the medial geniculate nucleus (MGN) and region dorsal to it. **(C’)** Taken from section adjacent to C double stained for *CNTNAP2* and GAD67 and counterstained blue with DAPI; *CNTNAP2* mRNA primarily associated with GAD67 immunonegative cells (arrows) and not GAD67+ cell cytoplasm (asterisks). **(D,E)** Represent mid thalamic regions and demonstrate widespread CNTNAP2 expression, with some areas of low expression such as the parafascicular complex (pF). (**D’,D”)** Taken from section adjacent to D double stained for *CNTNAP2* and FOXP2, showing that they can be co-expressed, or that expression can be mutually exclusive. **(F)** At more ventral levels, *CNTNAP2* expression highest laterally, posteriorly and close to third ventricle, but relatively weak medially and anteriorly. CM, centromedial nucleus; HIT, habenulo-interpeduncluar tract; LD, laterodorsal nucleus; MD, mediodorsal nucleus; VL, ventrolateral nucleus; VM, ventromedial nucleus; VP, ventroposterior nucleus. Scale bars = 1 mm **(A–F)**; 50 μm **(C’)**.

Figure 13A demonstrates that *CNTNAP2* was expressed at 8 PCW, being present in post-mitotic cells in the thalamic mantle but generally not expressed in the thalamic ventricular zone. This confirms supplementary data provided by Kim et al. (2023) in their human thalamus scRNAseq study suggesting localization to post-mitotic excitatory neurons at 4-8 PCW. It is also similar to what was observed in the ganglionic eminences and the cerebral cortex, with the exception of the cortical hem and thalamic eminence where expression was high (Figure 13A). In the thalamus, expression was highest medially, close to the ventricle, but this may simply reflect higher cell density.

A series of sections at 14 PCW, running from anterior and dorsal to more posterior and ventral, Figures 13B–F show that *CNTNAP2* expression, by the developmental stage when thalamic nuclei are emerging, no longer showed uniform expression across the thalamus in agreement with Ding et al. (2022). In the dorsalmost section (Figure 13B) habenular structures strongly expressed *CNTNAP2.* The pulvinar complex shows differential levels of staining in different areas. The medial pulvinar exhibited strong expression, as did more lateral regions of the lateral pulvinar. However, the inferior pulvinar exhibited relatively low expression. Moving more ventrally and anteriorly (Figure 13C) the habenula, medial pulvinar, LGN and the border with the pretectum all showed high expression, however, the MGN and an undefined region ventromedial to the MGN exhibited low expression. Figure 13C’ shows a high magnification image of a region at the edge of where multiple markers for GABAergic neurons are expressed at this level of the thalamus (Figures 12C,D). *CNTNAP2* mRNA expression is visualized as red dots predominantly around faint blue nuclei (arrowheads) and is not seen in conjunction with green immunofluorescence for GAD67+ GABAergic neurons (asterisks) suggesting *CNTNAP2* is predominantly expressed by glutamatergic neurons in the thalamus.

Moving further ventrally and posteriorly (Figures 13D,E) *CNTNAP2* exhibited widespread strong expression throughout the thalamus with the exception of the parafascicular nucleus and close to the 3rd ventricle (Figure 13D) in agreement with Ding et al. (2022). The CM-pF complex strongly expressed FOXP2 (Figures 8B) which is proposed to downregulate expression of *CNTNAP2* (Spiteri et al., 2007) so we double labeled an adjacent section for FOXP2 and *CNTNAP2* (Figures 13D’,D”). In the MGN, we observed double labeled cells in the more medial parts of the nucleus, but FOXP2+ only cells in lateral parts (Figure 13D’). The LGN exhibited *CNTNAP2* expression only, with no expression of *CNTNAP2* and FOXP2 in its most ventral part. We confirmed co-expression of *CNTNAP2* and FOXP2 in the CM, including within individual cells, but only FOXP2 was expressed in the pF, and only *CNTNAP2* was expressed lateral and dorsal to CM-pF. In the most posterior and ventral section (Figure 13E) expression of *CNTNAP2* was highest in ventral and posterior regions, and also close to the third ventricle.

Although FOXP2 has been proposed to downregulate *CNTNAP2* expression, we found that only in some FOXP2 expressing thalamic nuclei was *CNTNAP2* expression reduced, notably the pF and parts of the MGN. In the medial pulvinar, CM and parts of the MGN, co-expression was predominant.

## Discussion

**FIGURE 14:**
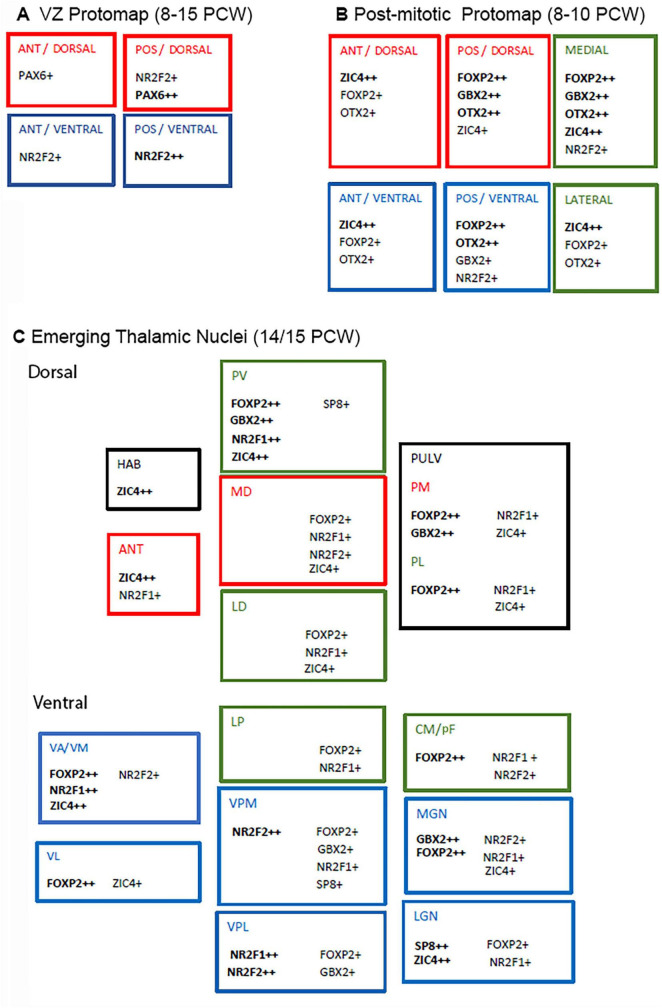
Summary diagram of transcription factor expression in the developing human thalamus. **(A)** Counter and overlapping gradients of PAX6 and NR2F2, suggesting at least 4 compartments that could produce different types of cells. **(B)** Illustrates protomap of transcription factor expression for post-mitotic neurons in early thalamus (8–10 PCW). For instance, ZIC4 tended to be more highly expressed anteriorly and laterally, FOXP2 posteriorly and medially. **(C)** By 14/15 PCW, emerging thalamic nuclei were distinguishable by their transcription factor expression. Higher order cognitive nuclei are marked in red, higher order sensory or motor integration nuclei in green and first order sensory or motor nuclei in blue (according to Sherman and Guillery, 2006). Each center characterized by its own combination of expression but no particular patterns are typical of each the three functional types. ANT, anterior; POS, posterior; HAB, habenula; PV; paraventricular; MD, mediodorsal; LD, laterodorsal; PULV; pulvinar; PM, medial pulvinar; PL, lateral pulvinar; VA/VM; ventral anterior/medial; VL; ventrolateral; LP, lateral posterior; VPM, ventral posterior medial; VPL, ventral posterior lateral; CM/PF, centromedian nucleus/parafascicular complex; MGN, medial geniculate nucleus; LGN, lateral geniculate nucleus.

We have provided the most comprehensive description to date of the molecular neuroanatomy of the human thalamus and associated structures between 8 and 16 PCW. A summary of transcription factor expression in both proliferative and post-mitotic compartments is provided in Figure 14. Our previous study showed that in primates, including human, thalamocortical fibers invade the cortical subplate almost as soon as it starts to form (8PCW in human) at an earlier developmental stage than in rodents (Alzu’bi et al., 2019). Here, we observed a relatively rapid transition from largely homogenous gene expression patterns in the thalamic mantle at 8 PCW to some evidence of differentiated patterns of expression by 10 PCW and recognizable thalamic nuclei by 14 PCW. In mouse, the equivalent time points would be E12.5, E14.5–15.5, and E18.5 (Gezelius et al., 2017; Suzuki-Hirano et al., 2010; Nakagawa, 2019) equivalent to 9 PCW, 13PCW, and 19 PCW in human (Workman et al., 2013). Thus, more evidence has been provided that human thalamic development shows a relatively accelerated time course compared to mouse. It has been demonstrated in mice that thalamic nucleus specific gene expression may be maintained by thalamocortical connectivity (Gezelius et al., 2017) and our observations suggest that earlier thalamocortical connectivity in human coincides with earlier thalamic nucleus formation.

### Protomap in the thalamic ventricular zone

There is an anterior ventral to posterior dorsal gradient of SHH concentration across the developing thalamus, as this morphogen is secreted from the anteriorly located ZLI and ventrally located basal plate (Kiecker and Lumsden, 2004; Nakagawa, 2019). SHH is known to sometimes suppress PAX6 expression in the forebrain (Chi et al., 2017) and so it was not surprising to observe higher PAX6 expression in the VZ posteriorly and dorsally, and absent from the ZLI. However, PAX6 was expressed strongly in the prethalamus of p3 which would be expected to receive a relatively high dose of SHH, indicating that co-expression and regulation of specific receptors and regulators of SHH signaling pathways in target cells is also important (Carballo et al., 2018). PAX6 expression was stronger and more persistent than that observed in mouse where weak expression dorsally was only observed up to E10.5 (Parish et al., 2016) the age of onset of neurogenesis in the mouse thalamus (Angevine, 1970). It is not clear why PAX6 expression extends throughout human thalamic neurogenesis which ranges from as early as 6PCW to at least 15 PCW (Rakic and Sidman, 1968; Alzu’bi et al., 2019; Workman et al., 2013).

We observed a different gradient of expression of NR2F2 in the thalamic VZ. Here expression was highest posteriorly and ventrally. Thus we have identified four zones to the human thalamic progenitor zone; anterior/ventral which is NR2F2+/PAX6-; anterior/dorsal which is NR2F2-/PAX6+; posterior/ventral which is NR2F2++/PAX6- and posterior/dorsal which is NR2F2+/PAX6++ (Figure 14A). Other transcription factors that might be predicted to display gradients of expression from mouse studies include OLIG2 expressed ventrally and DBX2 expressed dorsally (Vue et al., 2007). A more extensive future study of their expression patterns in human might be informative. We have observed very weak expression of OLIG2 immunoreactivity in or near the VZ, but unfortunately we only looked at 14 PCW. Cell lineage tracing studies in mouse have demonstrated that neuroprogenitors located in specific regions of the thalamic ventricular zone give birth to neurons destined to populate discrete thalamic nuclei (Shi et al., 2017). Thus anterior and dorsal thalamic VZ give rise to glutamatergic neurons that will populate anterior and medial higher order cognitive nuclei, whereas middle and ventral thalamic VZ populate higher order dorsolateral sensorimotor nuclei, and first order sensory nuclei located ventrally, posteriorly and laterally, respectively. It seems likely that a combinatorial protomap of transcription factor expression guides specification of thalamic neurons.

### The emergence of thalamic nuclei from protomaps of post-mitotic gene expression

Previous studies have shown that a variety of transcription factors can display universal expression in thalamic post-mitotic neurons, only to show restricted expression to specific nuclei as development proceeds. For instance, in mouse *Gbx2* is expressed by all thalamic neurons after leaving the cell cycle (Chen et al., 2009) but becomes restricted in expression to mostly medial and anterior nuclei, but also certain lateral and ventral nuclei posteriorly, in both mice and monkeys (Jones and Rubenstein, 2004). We have demonstrated that this also the case in human. GBX2 immunoreactivity became restricted to medial and posterior regions of the thalamus as early as 10 PCW, and by 14 PCW was confined to medial locations, the medial geniculate nucleus and other nearby posterior nuclei (Figures 4, 5, 10, 14B,C).

We observed some evidence of a gradient of increasing FOXP2 expression from anterior to posterior as early as 8 PCW, to also being widespread but stronger medially and posteriorly at 10 PCW, to being strongly expressed only in specific nuclei (e.g., CM, MGN, paraventricular thalamus) and absent from the anterior nuclei by 14 PCW (Figures 14B,C). An increasing expression from anterior to posterior was previously reported in mouse (Ebisu et al., 2017) but a medial to lateral gradient was not. Using transgenic mice expressing mutant *Foxp2*, Ebisu et al. (2017) demonstrated that FOXP2 directs development of posterior nuclei and interacts with GBX2 in counter gradients of expression. Posterior nuclei were smaller, while intermediate nuclei expanded along with thalamic territories expressing *Gbx2* and *Cadh6*. Anterior nuclei were unaffected. Our evidence suggests that something similar is occurring in human, although it is interesting that this interaction to form a protomap would be occurring in post-mitotic cells as these transcription factors are not highly expressed in ventricular zone progenitors, even at 8–10 PCW (Figures 2–5, 14B). From their spatial transcriptomic study at mid-gestation, Kim et al. (2023) reported two classes of thalamic excitatory neurons EN1 and EN2, with FOXP2 being a marker for EN2 which were enriched in higher order nuclei and posterior first order nuclei, such as the pulvinar, MD, ventral lateral nucleus, LGN, and MGN. This has been partially confirmed by our observations, although we observed only moderate FOXP2 expression in the LGN, and weak expression in more lateral parts of the MD.

Conversely, *ZIC4* expression exhibited a partially opposing gradient of expression at 8 PCW, being stronger anteriorly than posteriorly, and maintaining expression laterally where FOXP2 expression was weaker at 10 PCW (Figure 14B). By 14 PCW expression was confined to anterior and medial structures, but also the LGN (Figure 14C). According to supplementary data provide by Kim et al. (2023) *ZIC4* may be a marker for EN1 cells (see above) which were exclusively found in anterior nuclei but intermingled with EN2 cells in the LGN mid-gestation. In mouse, it is known that *Zic4* is preferentially expressed in LGN and *Foxp2* in MGN during post-natal development, and that they play roles in guiding development of visual and auditory pathways, respectively (Horng et al., 2009). Interestingly, it has been shown in mouse that expression of *Zic4* in progenitor cells with reduced *Pax6* expression gives rise to LGN neurons that maintain *Zic4* expression (Li et al., 2018). As we have shown that the human thalamus also has a ventral and posterior *ZIC4+* PAX6- domain early in development, we can also surmise that this might be the location for production of LGN neurons, as has been demonstrated in mouse by cell lineage tracing studies (Shi et al., 2017). However, human seems to differ significantly from mouse in not expressing *ZIC4* as strongly in the prethalamus (Li et al., 2018).

NR2F1, NR2F2, and SP8 also exhibited varying levels of expression across the thalamus. Although it was possible to attribute different combinations of transcription factor expression to different emerging nuclei and detect counter gradients of expression; these patterns revealed more about the location of the nuclei rather than predicting their future functional roles in cognition, sensory and motor integration or as first order relay nuclei (Figure 14C).

### SP8 expression in the thalamus

Detection of SP8 immunoreactivity in the thalamus was a surprising finding. This may be non-specific staining. The antibody has been characterized by protein array assay (only one interaction peak, the antigen, but not by Western blot).^4^ However, the staining patterns observed mostly concurred with specific and expected patterns of staining in human fetal telencephalon achieved with a different antibody (now unavailable; Alzu’bi et al., 2017a). We employed the new antibody as a marker of GABAergic neurons originating either from the prethalamus or caudal ganglionic eminence (CGE). We did observe strong SP8-like immunoreactivity in the prethalamus at 8PCW, but careful observation found SP8+ cells within the posterior and ventral margins of the thalamus in particular. These may have migrated into the thalamus from the prethalamus, however, we found the largest concentration posteriorly close to the ventral border with the SP8 negative pretectum, suggesting they could have been generated in the thalamus.

At 14 PCW SP8 immunoreactivity was a strong marker for the reticular formation which develops from the prethalamus. However, it was also a reliable marker for LGN but not the MGN. Potentially, the group of SP8+ cells observed posteriorly at 8PCW could have been the nascent LGN, although its location appears too ventral for this developmental stage. SP8 immunoreactivity showed strong co-localization with GBX2 at the cellular level in the anterior medial thalamus, but in posterior regions it was co-expressed in the same nuclei but not so much in the same cells. We have provided evidence for a potential novel role for SP8 in thalamic development, specifying the differentiation of distinct thalamic nuclei in concert with other transcription factors. This may be human specific as it has not been previously reported in other studies in other species.

### Invasion of the thalamus by GABAergic interneurons

It is known that in the primate thalamus there is a far higher proportion of GABAergic interneurons found within the nuclei, as opposed to thalamic reticular neurons that provide inhibition to thalamic neurons from a location outside of the thalamus (Braak and Weinel, 1985; Hunt et al., 1991; Arcelli et al., 1997). In both rodents and primates, the source of these interneurons has been demonstrated as being either the rostral midbrain for those expressing SOX14 and OTX2 (80% in rodents, 90% in marmoset) or from forebrain, using cell tracing studies (Jager et al., 2021) but not from the dorsal thalamic ventricular zone in either rodents or primates.

In rodents, the forebrain origin of GABAergic interneurons is the prethalamic (p3) progenitor zone (Jager et al., 2021) although the rostralmost thalamic ventricular zone may give rise to GABAergic projection neurons of the intergeniculate leaflet and ventral LGN (Vue et al., 2007; Delogu et al., 2012). This is also the case in marmosets but in human, it has long be argued that late born thalamic neurons can derive from the medial CGE (Letinic and Rakic, 2001) a source of SP8 and NR2F2 expressing GABAergic neurons in the telencephalon (Alzu’bi et al., 2017a; Alzu’bi et al., 2017b) and this has been recently confirmed by transcriptomic studies of cell lineage (Bakken et al., 2021; Kim et al., 2023). However, *in situ* hybridization studies have demonstrated a complete absence of expression of *FOXG1*, a marker of telencephalic neurons, in the thalamus at 15 PCW (Ding et al., 2022) suggesting these neurons arrive later in development.

We have demonstrated that *SOX14*, OTX2 and GAD67 expressing cells appear in the posterior two thirds of the thalamus by 14 PCW. It has previously been observed that there is a posterior to anterior appearance of interneurons across the thalamus in non-human primates and carnivores (Jones, 2002) and that interneurons arrive late in the thalamus; mid-gestation in monkeys and at birth in ferrets (Jones, 2002). In human a ScRNAseq/spatial transcriptomic study has identified a class of *SOX14+/*OTX2+ GABAergic neurons derived from the midbrain that appear in the thalamus after 10 PCW and inhabit the entire thalamus by mid gestation, but only selected nuclei at 14 PCW (Kim et al., 2023). Our observations confirm that appearance of interneurons in the thalamus proceeds posterior to anterior, is co-incident with the emergence of thalamic nuclei, but again stresses the point that human thalamic development is taking place relatively earlier than in other species.

We found no evidence for interneurons progressing much further anteriorly between 14 and 16 PCW, which leaves open the question as to whether anterior nuclei are populated by midbrain origin GABAergic neurons much later in development, or whether these nuclei are populated by cells of prethalamic or telencephalic origin. The anterior nuclei have the highest proportion of interneurons in humans and macaques (Hunt et al., 1991; Dixon and Harper, 2001; Popken et al., 2002) and this may be achieved by supplementation from the CGE. On the other hand, whereas in rodents midbrain origin interneurons preferentially cluster in the posterior and lateral nuclei and forebrain origin neurons in the anterior and medial (Jager et al., 2021) analysis of non-human primates and human data suggests a more homogenous distribution of the two interneuron classes across the thalamic nuclei (Jager et al., 2021; Bakken et al., 2021; Kim et al., 2023). Making some pause in the migration a more likely explanation.

Although *SOX14+* presumed GABAergic interneuron precursors were spread throughout the posterior two thirds of the thalamus at 14 PCW, a large proportion did not co-express GAD67, suggesting that they were not functionally mature as GAD67 expression is activity dependent (Lau and Murthy, 2012). However, the CM-pF showed precocious expression of GAD67. This suggests these nuclei may mature more quickly than others. In the adult brain, the CM receives both multimodal sensory input and afferents from the ascending reticular activating system, while projecting primarily to sensorimotor cortical areas, however, it connects indirectly via other thalamic nuclei with a large proportion of the forebrain and is thus proposed to play a role in arousal and attention (Jang et al., 2014; llyas et al., 2019; Van der Werf et al., 2002; Kinomura et al., 1996). In development, thalamocortical innervation occurs first in the subplate of the sensorimotor cortex (Alzu’bi et al., 2019; Krsnik et al., 2017) and projections from the CM-pF may initiate early co-ordinated activity in thalamus and cortical subplate that drives development (Molnár et al., 2020; Molnár et al., 2023).

### Expression of a neurodevelopmental disease susceptibility gene in developing thalamus

*CNTNAP2* expression was uniform across the thalamus at 8 PCW, as was the case for a number of genes and proteins we examined at this stage. This suggests that CNTNAP2 plays a significant role in thalamic development, but that different regions of the thalamus are not yet following distinct developmental pathways. By 14 PCW, *CNTNAP2* was found to be expressed by glutamatergic, not GABAergic, neurons and its expression varied between the different thalamic nuclei. Broadly speaking, this partially confirmed our hypothesis that this neurodevelopmental susceptibility gene would be expressed in higher order thalamic nuclei involved in cognitive processes, showing strong expression in the medial pulvinar, MD and laterodorsal nucleus, for instance. However, expression was also detected in the LGN, as well as ventrolateral regions, demonstrating it can be expressed by first order thalamic centers.

FOXP2 negatively regulates expression of *CNTNAP2* (Spiteri et al., 2007; Vernes et al., 2007) providing a possible explanation as to why mutations in both genes disrupts language development (Lai et al., 2001; Whitehouse et al., 2011). It might be predicted that thalamic nuclei that strongly express FOXP2 would fail to express CNTNAP2, but we did not find this always to be the case. The centromedian nucleus (CM) in particular, showed strong expression of both, suggesting that FOXP2 regulation of *CNTNAP2* expression is context specific. Intriguingly, three thalamic regions primarily shown to suffer reductions in volume in schizophrenia patients, the MD, pulvinar and CM (Kemether et al., 2003) all highly expressed both *CNTNAP2* and FOXP2. It has been suggested that evolution of language acquisition, including expansion of the pulvinar, could be driven by the human specific isoform of FOXP2 (Ebisu et al., 2017). Perhaps the ability to negatively or positively regulate expression of *CNTNAP2* is crucial to this process.

CNTNAP2 is robustly expressed in the ganglionic eminences and the neurons they produce, i.e. GABAergic basal ganglion neurons and cortical interneurons (Peñagarikano et al., 2011; Gordon et al., 2016; Ding et al., 2022) where it is proposed to play roles in neurogenesis, neurite growth and cell migration (Peñagarikano et al., 2011; Gao et al., 2018; Hali et al., 2020). However, in the thalamus expression was not found in GAD67+ cells but in presumptive GAD67- glutamatergic neurons. It was not found to be expressed in the ventricular zone, suggesting it does not play a role in neurogenesis in the thalamus, although it was highly expressed in the VZ of the epithalamus/habenula. As the habenula is considered to play a crucial role in regulating negatively motivated behavior and is implicated in several psychiatric illnesses including major depression (Hu et al., 2020) a better understanding of its development and expression of susceptibility genes would be worth pursuing. In the thalamus, CNTNAP2 may still play crucial roles in directing migration of thalamic neuroblasts to the correct thalamic location, and in directing axon outgrowth.

## Conclusion

Even though the thalamus has co-evolved with the cerebral cortex, and is implicated in many neurodevelopmental disorders of higher cognition, study of the development of specifically the human thalamus has lagged behind other brain regions. This study helps to address that and demonstrates many similarities between human development and animal models, but at the same time highlights the extent to which some features of human thalamus develop relatively quickly, for instance emergence of distinct nuclei. On the other hand, the migration of GABAergic neurons into the thalamus occurs over a protracted period with forebrain derived neurons arriving later than the midbrain derived contingent. We have identified SP8 expression in some developing thalamic nuclei as a potentially human specific trait. Combined with scRNAseq data, it should be possible in the future to use these findings to help interpret expression patterns of neurodevelopmental disease susceptibility genes in the thalamus and generate hypotheses as to how disruption of their expression leads to disordered thalamic development and function.

## Data Availability

The original contributions presented in the study are included in the article/supplementary material, further inquiries can be directed to the corresponding author.

## Footnotes

1
http://www.hdbr.org

2
https://www.hdbr.org/uploads/default/factsheets/fixation_and_sectioning.pdf

3
https://hdbratlas.org/fetal-stages/13pcw.html

4
https://www.proteinatlas.org/ENSG00000164651-SP8/summary/antibody#PA
